# Relative catalytic efficiencies and transcript levels of three d‐ and two l‐lactate dehydrogenases for optically pure d‐lactate production in *Sporolactobacillus inulinus*

**DOI:** 10.1002/mbo3.704

**Published:** 2018-08-01

**Authors:** Bin Wu, Qi Yu, Shan Zheng, Marcelo Monteiro Pedroso, Luke W. Guddat, Bingfang He, Gerhard Schenk

**Affiliations:** ^1^ College of Biotechnology and Pharmaceutical Engineering Nanjing Tech University Nanjing China; ^2^ School of Chemistry and Molecular Biosciences The University of Queensland Brisbane Australia; ^3^ School of Pharmaceutical Sciences Nanjing Tech University Nanjing China

**Keywords:** d‐lactate, d‐lactate dehydrogenase, l‐lactate dehydrogenase, optically pure, *Sporolactobacillus inulinus*

## Abstract

As the optical purity of the lactate monomer is pivotal for polymerization, the production of optically pure d‐lactate is of significant importance. *Sporolactobacillus inulinus* YBS1‐5 is a superior optically pure d‐lactate‐producing bacterium. However, little is known about the relationship between lactate dehydrogenases in *S. inulinus* YBS1‐5 and the optical purity of d‐lactate. Three potential d‐lactate dehydrogenase (D‐LDH1‐3)‐ and two putative l‐lactate dehydrogenase (L‐LDH1‐2)‐encoding genes were cloned from the YBS1‐5 strain and expressed in *Escherichia coli* D‐LDH1 exhibited the highest catalytic efficiency toward pyruvate, whereas two L‐LDHs showed low catalytic efficiency. Different neutralizers significantly affected the optical purity of d‐lactate produced by strain YBS1‐5 as well as the transcription levels of *ldh*Ds and *ldh*Ls. The high catalytic efficiency of D‐LDH1 and elevated *ldh*D1 mRNA levels suggest that this enzyme is essential for d‐lactate synthesis in *S. inulinus* YBS1‐5. The correlation between the optical purity of d‐lactate and transcription levels of *ldh*L1 in the case of different neutralizers indicate that *ldh*L1 is a key factor affecting the optical purity of d‐lactate in *S. inulinus* YBS1‐5.

## 1 INTRODUCTION

Polylactic acid (PLA), an attractive versatile biodegradable plastic, has great potential to replace petroleum‐based plastics (Abdel‐Rahman & Sonomoto, 2016; Singhvi, Gurjar, Gupta, & Gokhale, 2015). Currently, around 450 million kilograms of PLA are produced annually worldwide, representing almost one quarter of the global plastic production; this figure is steadily increasing (Wang, Tashiro, & Sonomoto, 2015). However, conventional PLA is produced from optically pure l‐lactate, and a low melting point limits its application. It was reported that a PLA stereo‐complex obtained by blending L‐PLA and D‐PLA had higher thermal resistance, mechanical performance, and hydrolysis resistance compared with the respective single polymers, thus representing an attractive option for improving the performance of conventional l‐lactate polymers (Tsuji, 2005). In this strategy, the production of optically pure lactic acid has been boosted, which is required for lactic acid polymerization (Li, Wang, Ju, Yu, & Ma, 2013; van Wouwe, Dusselier, Vanleeuw, & Sels, 2016).

To date, almost all lactate with high optical purity is manufactured by microbial fermentation using lactic acid bacteria (LAB) and some genetically modified strains such as *Escherichia coli* and *Saccharomyces cerevisiae* (Baek, Kwon, Kim, & Hahn, 2016; Baek et al., 2017; Li, Sun, Wu, & He, 2017; Othman, Ariff, Rios‐Solis, & Halim, 2016). The fermentative metabolism of LAB is characterized by the glycolytic breakdown of carbohydrates, comprising the conversion of pyruvate into lactate as the last step (Mohamed, Yukihiro, & Kenji, 2013). In this pathway, lactate dehydrogenases (LDHs) play key roles by catalyzing not only the transformation of pyruvate to lactate, but also the oxidation of nicotinamide adenine dinucleotide (NADH), which constitutes an important step in the metabolism and energy conversion of living cells (Andreevskaya et al., 2015; Wang, Ingram, & Shanmugam, 2011). Two optically pure isomers of lactate were produced from pyruvate as separate entities through reactions catalyzed by either the chiral‐specific d‐lactate dehydrogenase (D‐LDH, EC 1.1.1.28) or l‐lactate dehydrogenase (L‐LDH, EC 1.1.1.27) (Singhvi, Jadhav, & Gokhale, 2013; Sun, Zhang, Lyu, Wang, & Yu, 2016; Wang, Cai, Zhu, Guo, & Yu, 2014; Zheng et al., 2012; Zhu et al., 2015). A sequence comparison has shown that D‐LDH and L‐LDH belong to two distinct families, the NAD‐dependent L‐ and D‐2‐hydroxyacid dehydrogenases, respectively (Cristescu & Egbosima, 2009; Ma et al., 2007). Almost all LAB contains both L‐LDH‐ and D‐LDH‐encoding genes (*ldh*L*, ldh*D), however, the optical purity for lactate produced by various LAB strains differs significantly, and in some cases even completely opposite (Zheng et al., 2012). In addition, fermentation conditions might influence the optical purity of lactate. For instance, Fukushima and coworkers reported that the optical purity of d‐lactate from *Lactobacillus delbrueckii* LD2008 was affected by the carbon sources used (Fukushima, Sogo, Miura, & Kimura, 2004). However, studies on the associations of L‐ and D‐LDH with the optical purity of lactate are scarce. In a study by Zheng et al., three *Lactobacillus* strains, *L. delbrueckii* ATCC 11842 (D‐LAB strain), *L. plantarum* ATCC 14917 (DL‐LAB strain), and *L. casei* (L‐LAB strain), were selected to demonstrate that lactate was produced with different optical purities. They found that L‐ and D‐LDHs coexisted in three *Lactobacillus* genomes, with the relative catalytic efficiencies of enzymes playing a crucial role for the optical purity of lactate in *Lactobacillus* strains (Zheng et al., 2012). Subsequently, a study with *Bacillus coagulans* 2–6, which contained both D‐LDH and L‐LDH, showed that elevated catalytic efficiency of L‐LDH toward pyruvate and high transcription ratio of *ldh*L to *ldh*D constituted the main factors for the high optical purity of l‐lactate produced by this strain(Sun et al., 2016; Wang et al., 2014). These findings indicate that different L‐ and D‐LDH activity levels and *ldh*L and *ldh*D mRNA amounts in LAB contribute to differences in the ratio of the two isomers.

Compared to l‐lactate production, biotechnological tools for the commercial manufacturing of d‐lactate have yet to be developed. Several microbial strains have been described as homo‐fermentative d‐lactate producers, including *Sporolactobacillus inulinus, Sporolactobacillus laevolacticus, Lactobacillus delbrueckii, Lactobacillus lactis*, metabolically engineered *Saccharomyces cerevisiae,* and *Escherichia coli* (Baek et al., 2017; Li et al., 2013; Othman et al., 2016; Singhvi et al., 2013; Wang et al., 2011, 2015). Among them, *S. inulinus* has been considered as a superior candidate for the industrial production of optically pure d‐lactate, and considerable efforts has been deployed to enhance the efficiency and cost‐effectiveness of *S. inulinus* d‐lactate production (Li et al., 2017; Wang et al., 2011). On the other hand, the complete genome sequence of *S. inulinus* CASD was reported in 2010 (Yu et al., 2011). Meanwhile, a D‐LDH from this strain that could preferentially use both NADH and NADPH (nicotinamide adenine dinucleotide phosphate) as coenzymes was cloned and named DLDH744 (Zhu et al., 2015). More recent studies by Zhu, Xu, Wang, Dong, and Yu (2015) confirmed that the above enzyme had both D‐LDH and glutamate dehydrogenase activities. However, the connections of *S. inulinus* LDHs to the optical purity of d‐lactate has remained undefined.

In our previous study, we generated a mutant of *S. inulinus,* strain YBS1‐5 with high titer and optical purity of d‐lactate, by combining two physical mutation methods, low‐energy ion implantation treatment as well as atmospheric and room temperature treatment (Bai, Gao, Sun, Wu, & He, 2016). During the fermentation optimization of strain YBS1‐5, we found that the final optical purity of d‐lactate is affected by the neutralizer used (Zheng, Liu, Sun, Wu, & He, 2017). Indeed, NaOH was shown to increase d‐lactate productivity, while reducing the optical purity of this product (Zheng, Bai, Xu, & He, 2012; Zheng, Xu, Bai, & He, 2014; Zheng et al., 2017). In this study, based on the whole‐genome sequence of *S. inulinus* CASD, three potential D‐LDH‐ and two L‐LDH‐encoding genes (*ldh*Ds, *ldh*Ls), as well as a l‐lactate permease‐encoding gene (*lld*P) that involved in l‐lactate utilization were annotated from the whole‐genome sequence of *S. inulinus* YBS1‐5. To systematically assess the associations of these five enzymes with the optical purity of d‐lactate, their functional expression in *E. coli* was carried out to biochemically characterize them. Next, phylogenetic trees were constructed to investigate their evolution at amino acid level. Finally, the relative transcription levels of *S. inulinus* YBS1‐5 *ldh*Ds, *ldh*Ls, and *lld*P under different neutralizers were quantified, and the effects of *ldh*s and *lld*P transcriptional levels on optical purity of d‐lactate produced by *S. inulinus* YBS1‐5 were analyzed.

## 2 MATERIALS AND METHODS

### 2.1 Strains, plasmids, media, and chemicals

*Sporolactobacillus inulinus* YBS1‐5 (deposited in the China Center for Type Culture Collection (CCTCC) with an accession number of M2012516) was used as a source for cloning of D‐ and L‐LDH‐encoding genes as well as for d‐lactate production. The fermentation medium contained the following ingredients (g/L): glucose 150, yeast extract 5, MgSO_4_ 0.5, KH_2_PO_4_ 1. Solid CaCO_3_ (90 g/L) or NaOH, KOH, and NH_4_OH solutions at 10 mmol/L was used for pH control during the fermentation process, respectively. *E. coli* DH5α was used for cloning, and *E. coli* BL21 (DE3) for protein expression. *E. coli* strains were grown in Luria‐Bertani (LB) medium on a rotary shaker at 37°C. Plasmid pMD18‐T was used for cloning the genes encoding LDHs, whereas plasmid pET‐28a was used to express the recombinant LDHs with a N‐terminal His‐tag (Sun et al., 2016; Zhou, Zhang, Meng, Zhang, & Li, 2014). All the reagents were of analytical grade and commercially available.

### 2.2 Gene cloning, expression, and purification of *S. inulinus* YBS1‐5 LDHs

Each of the *ldh*L and *ldh*D genes were amplified from *S. inulinus* YBS1‐5 genomic DNA by PCR. Based on the whole‐genome sequence of *S. inulinus* CASD (GenBank accession no. AFVQ00000000), primers were designed from three D‐LDHs and two L‐LDHs of *S. inulinus* CASD, released in GenBank under accession numbers KLI01297.1 (D‐LDH), KLI03733.1 (D‐LDH), KLI03581.1 (D‐LDH), and KLI02950.1 (L‐LDH), WP010025461 (L‐LDH), and introduced *NheI* and *HindIII* restriction sites (for D‐LDH1), *NdeI* and *HindIII* restriction sites (for D‐LDH2/3 and L‐LDH1/2) and a recognition site for enterokinase that was used to remove His‐tag from recombinant LDHs by enterokinase (Table S1). The amplified DNA products were ligated into the pMD18T vector for sequencing. After verification of the amplification fidelity, the digested DNA fragments were subcloned into the pET28a plasmid. The resulting recombinant plasmids were named pET‐*ldh*D1, pET‐*ldh*D2, pET‐*ldh*D3, pET‐*ldh*L1, and pET‐*ldh*L2, respectively, and transformed into *E. coli* BL21 for protein expression.

*Escherichia coli* BL21 cells harboring the recombinant plasmid were incubated in LB medium at 37°C. At an optical density at 600 nm (OD600) of 0.6–0.8, isopropyl‐β‐D‐thiogalactopyranoside (IPTG) at 0.1 mmol/L was added to induce gene expression, and growth was continued at 20°C for another 12 hr. The cells were harvested by centrifugation, resuspended in 50 mmol/L Tris‐HCl buffer (pH 7.5), and disrupted by sonication (300 W, pulse on 3 s, pulse off 5 s) on ice for 5 min using SCIENTZ YJ96‐IIN sonicator (Ningbo Xinzhi Biotechnology Co., Ltd, China). Crude lysates were obtained for protein purification by removing cell debris with centrifugation at 4°C. LDHs were purified by nickel affinity chromatography according to the manufacturer's instructions (GE Healthcare). N‐terminal His‐tagged LDHs were cleaved by recombinant enterokinase and purified as the method described by Zheng et al. (2017). Apparent molecular mass and purity of the five LDHs were monitored by sodium dodecyl sulfate–polyacrylamide gel electrophoresis (SDS‐PAGE). Protein concentrations were determined by the Bradford method using bovine serum albumin (BSA) as standard (Bradford, 1976).

### 2.3 Enzymatic activity assays of *S. inulinus* YBS1‐5 LDHs

The activities of purified LDHs were assayed by monitoring the decrease in absorption at 340 nm (from NADH) with pyruvate as substrate, as described elsewhere (Zhu et al., 2015, 2015). The reaction was initiated by enzyme addition. One unit of activity is defined as the amount of enzyme converting 1 μmol NADH per minute under standard conditions.

### 2.4 Biochemical characterization of *S. inulinus* YBS1‐5 LDHs

The optimal pH of purified LDHs was determined using a range of different buffer systems, including MES/NaOH (pH 5.0–7.0), Tris‐HCl (pH 7.0–9.0), and glycine/NaOH (pH 9.0–10.5), at a concentration of 50 mmol/L each. The temperature optimum of LDHs was assessed over the range from 20°C to 50°C.

A kinetic study of D‐LDH1 was performed at pH 6.0 and 35°C. For D‐LDH2 and D‐LDH3, the assay pH was 7.5 and 5.5, respectively, whereas the assay temperature was 30°C. The kinetic parameters of L‐LDH1 and L‐LDH2 were determined at pH 7.0 at 45°C and 40°C, respectively. In these experiments, NADH was used as the cofactor at a constant concentration of 0.4 mmol/L. Pyruvate concentrations ranged from 0.05 to 40 mmol/L. Kinetic parameters (i.e., *K*_m_ and *V*_max_) were determined using nonlinear least square regression with Origin 8.0 software.

The effects of different metal ions (Ca^2+^, Mg^2+^, Fe^3+^, Na^+^, and K^+^) on the enzymatic activities of D‐LDHs and L‐LDHs were investigated by separately preincubating the enzyme aliquots with these ions at a final concentration of 5 mmol/L for 1 hr.

To investigate the substrate specificity of three D‐LDHs and two L‐LDHs, glyoxylate, pyruvate, 2‐ketobutyrate, 2‐ketovalerate, 2‐ketoisocaproate, and phenylpyruvate were tested as substrates. The enzyme activities toward these substrates were measured using reaction conditions as described above.

### 2.5 Phylogenetic analysis of LDHs

For phylogenetic analysis, we first performed online BLASTN and BLASTP searches in BLAST (http://blast.ncbi.nlm.nih.gov/blast.cgi) based on the amino acid sequences of D‐LDH1, D‐LDH2, and D‐LDH3 to obtain other D‐LDH amino acid sequences with high sequence similarity. Then, two key terms, D‐LDH and bacterial name of common D‐LDH producing strains, were used to carry out GenBank advanced search (http://www.ncbi.nlm.gov), and sequences belonging to D‐hydroxyacid dehydrogenase, D‐glycerate dehydrogenase, D‐phosphoglycerate dehydrogenase, D‐hydroxyisocaproate dehydrogenase, formate dehydrogenase, and malic dehydrogenase in different species were selected for phylogenetic analysis (Table S2). In the same manner, a series of amino acid sequences associated with L‐LDH1 and L‐LDH2 were obtained from different species (Table S3). Multiple sequence alignment was performed by ClustalX V1.8. Phylogenetic analysis was conducted using MEGA6 software with full‐length amino acid sequences. The neighbor‐joining method (NJ) was used for phylogenetic tree generation (Cristescu & Egbosima, 2009; Zheng et al., 2012; Zhu et al., 2015).

### 2.6 d‐lactate production under different neutralizing agents in a 7.0‐L bioreactor

The effects of neutralizing agents on d‐lactate production by *S. inulinus* YBS1‐5 were assessed using NaOH, KOH, and NH_4_OH as neutralizers, respectively. Batch fermentation was carried out at 37°C and 120 rpm in a 7.0‐L bioreactor (Winpact, Major Science, USA) containing 3 L fermentation medium. During the fermentation process, the broth pH was automatically maintained at 6.5 by the addition of 10 mol/L of NaOH, KOH, or NH_4_OH. CaCO_3_ was used as a control neutralizer. All fermentation samples were taken out for analyzing d‐lactate concentration and optical purity by high‐performance liquid chromatography (HPLC) using a UV detector (254 nm) with a chiral column (150 mm × 4.6 mm, SUMICHIRALOA‐5000) as described previously (Bai et al., 2016; Wang et al., 2011). The optical purity of d‐lactate was defined as [(d‐lactate − l‐lactate)/( d‐lactate + l‐lactate)] × 100%. Cell growth was measured at a wave‐length of 660 nm.

### 2.7 Real‐time PCR transcript quantification

Quantitative real‐time PCR was employed to determine the transcription levels of genes encoding five LDHs in presence of different neutralizing agents. According to the whole‐genome sequence of *S. inulinus* CASD, two l‐lactate permeases (No. KLI02332.1 and WP_047035140.1) were present. However, a sequence alignment revealed that these two enzymes shared 100% sequence identity. It was thus possible that a l‐lactate permease‐encoding gene was also present in *S. inulinus* YBS1‐5. Therefore, the transcription levels of l‐lactate permease‐encoding gene in presence of different neutralizers were also measured. Cells were harvested in the logarithmic phase by centrifugation (8,000*g* for 10 min, 4°C) for RNA isolation with an E.Z.N.A bacterial RNA kit (Omega). Sampling time was 50 and 56 hr with CaCO_3_ as neutralizer, and 32 and 40 hr when using NaOH, KOH, or NH_4_OH. Total RNA amounts were determined by absorbance measurements at 260 nm. Gene‐specific PCR primers were designed with Beacon Designer software (Table S4). Quantitative real‐time PCR reactions were performed on a SteponePlus^™^ real‐time PCR system (ABI) with SYBR Premix Ex Tag (TaKaRa, China) according to the manufacturer's instructions. The 2^−∆∆Ct^ relative quantification method was used to assess mRNA levels, with 16S rRNA as the internal reference gene (Sun et al., 2016; Wang et al., 2014; Zheng et al., 2012).

## 3 RESULTS AND DISCUSSION

### 3.1 Cloning, expression, and purification of LDHs from *S. inulinus* YBS1‐5

Based on the genome sequence of *S. inulinus* CASD (GenBank accession no. AVFQ00000000), three possible D‐LDH and two L‐LDH‐encoding genes were cloned from *S. inulinus* YBS1‐5, and named *ldh*D1, *ldh*D2, *ldh*D3, and *ldh*L1, *ldh*L2, respectively. The *ldh*D1 and *ldh*D2 genes are 1,005 bp and 1,026 bp, encoding 334 (36.7 kDa) and 341 (37.2 kDa) amino acids, respectively. D‐LDH3 is a 335‐amino acid enzyme with a calculated molecular mass of 36.6 kDa. *S. inulinus* YBS1‐5 L‐LDH1 and L‐LDH2 consist of 326 and 311 residues with calculated molecular masses of 35.5 kDa and 34.4 kDa, respectively. The amino acid sequences of these five enzymes share 100% identity with putative D‐LDH (KLI01297.1), putative D‐LDH (KLI03733.1), validated D‐LDH (KLI03581.1), putative L‐LDH (KLI02950.1), and putative L‐LDH (WP_010025461). However, they exhibit lower sequence similarities to other D‐LDHs/L‐LDHs from different species. For instance, D‐LDH1 shares 55% and 47% identities to D‐LDH from *Clostridium botulinum* (WP_045905752.1) and *Lactobacillus parabuchnei* (WP_057910218). D‐LDH2 shares 61% and 57% identity, respectively, with D‐LDH from *Streptococcus* sp. DD13 (WP_067100797.1) and *Lactobacillus curvatus* (WP_065825558.1). D‐LDH3 showed 62%–65% amino acid identities with D‐LDHs from different *Lactobacillus* species. Similar results were observed for L‐LDH1 and L‐LDH2.

Five recombinant enzymes were expressed in *E. coli* and purified to homogeneity on a Ni^2+^‐charged column, and the N‐terminal His tag was subsequently removed by His‐tagged rEK cleavage. The purified enzymes appeared each as a single band on SDS‐PAGE, and apparent subunit molecular weights were in agreement with the respective calculated molecular masses (Figure S1). D‐LDH3 also shares 100% identity with DLDH744 from *S. inulinus* CASD. Zhu et al. found a molecular mass of approximately 37 kDa for DLDH744 enzyme, as determined by SDS‐PAGE, whereas native PAGE analysis indicated a molecular weight of approximately 70 kDa (Zhu et al., 2015, 2015). The authors thus concluded that DLDH744 existed as a homodimer. Therefore, we hypothesize that D‐LDH3 may also be a homodimeric protein. To date, expression and characterization of the remaining four enzymes have not been reported. In general, reports in BRENDA (http://www.brenda-enzymes.info) indicate that most LDHs may form homodimers.

### 3.2 Biochemical properties of *S. inulinus* YBS1‐5 D‐LDHs

**Figure 1:**
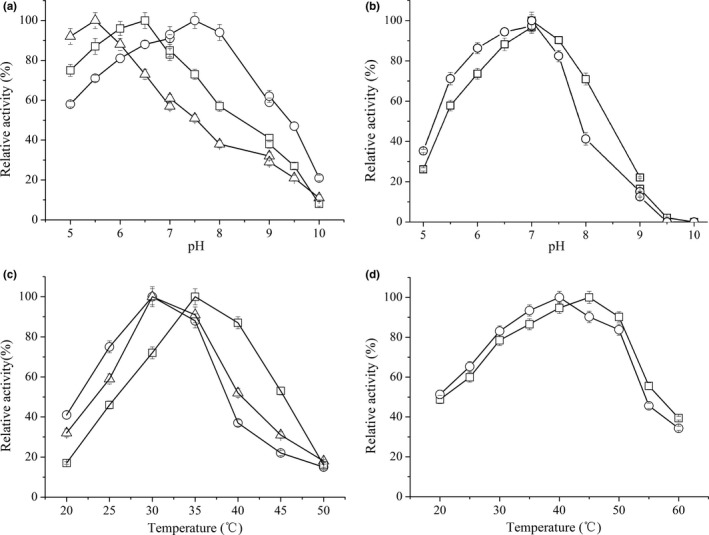
Effect of pH (a, b) and temperature (c, d) on the activity of D‐LDHs and L‐LDHs. Symbols represent: (a), D‐LDH1 (square), D‐LDH2 (circle), and D‐LDH3 (triangle); (b), L‐LDH1 (square), L‐LDH2 (circle); (c), D‐LDH1 (square), D‐LDH2 (circle), and D‐LDH3 (triangle); (d), L‐LDH1 (square), L‐LDH2 (circle). Citric acid‐citrate sodium (C_6_H_8_O_7_–Na_3_C_6_H_5_O_7_) buffer, pH 5.0–7.0, Tris‐HCl buffer, pH 7.0–9.0, Glycine‐NaOH buffer, pH 9.0–10.0. Errors bars represent the standard deviations of the means from three independent experiments

D‐LDH1, D‐LDH2, and D‐LDH3 enzymes consistently exhibited marked catalytic activity toward pyruvate with NADH as the cofactor. The specific activities of purified enzymes were 51.4 U/mg for D‐LDH1, 39.5 U/mg for D‐LDH2, and 7.0 U/mg for D‐LDH3. However, the specific activities of L‐LDH1 and L‐LDH2 with pyruvate as the substrate were only 0.71 and 0.58 U/mg, that is, much lower than those of three D‐LDHs. Zhu et al. (2015) reported the specific activity of DLDH744 to be 7.5 U/mg which is similar to that of D‐LDH3. Consistent with DLDH744 data, D‐LDH3 also had an optimum pH of 5.5. The optimal pH for D‐LDH1 and D‐LDH2 were 6.5 and 7.5, respectively (Figure 1a). D‐LDHs from other species, such as *Lactobacillus jensenii*,* Acetobacter aceti*, and *L. delbrueckii*, had a more alkaline optimal pH (7.4–8.5) (Kim, Gu, Kim, & Kim, 2014; Min, Yeon, Um, & Kim, 2016; Zheng et al., 2012). The pH profile for purified L‐LDH1 and L‐LDH2 was shown in Figure 1b. Their optimal pH of two L‐LDHs was found to be 7.0, similar to that of D‐LDH1 and D‐LDH2. Cook, Senkovich, Hernandez, Speed, and Chattopadhyay (2015) reported that L‐LDH of *Cryptosporidium parvum* had an optimum pH of 5.0–5.5. L‐LDH from *B. coagulans* was reported to have a more acidic pH optimum (pH 4.0) (Jiang, Xu, Sun, Zheng, & Ouyang, 2014). Three D‐LDHs showed similar activity‐temperature profiles, and optimal temperatures were 30°C–35°C (Figure 1c). Low optimal temperatures for D‐LDHs from *L. delbrueckii, L. jensenii,* and *Staphylococcus* sp. were also observed (Isobe, Koide, Yokoe, & Wakao, 2002; Kim et al., 2014; Zheng et al., 2012). However, maximal activity of L‐LDH1 and L‐LDH2 was observed at 45°C and 40°C, respectively (Figure 1d). The results indicate that D‐LDHs in *S. inulinus* YBS1‐5 have lower optimal temperature than L‐LDHs. In general, optimal temperatures of L‐LDHs have been reported to be higher than those of D‐LDHs in same host (Jun et al., 2013). In this respect, an elevated culture temperature may induce a decline in optical purity of d‐lactate, which has frequently been observed (Abdel‐Rahman & Sonomoto, 2016; Gu et al., 2014). For instance, Gu et al. (2014) reported a dramatic drop in the optical purity of d‐lactate produced by *Lb. coryniforms* KCTCC 3535 at temperature greater than 40°C, whereas d‐lactate is the dominant product at 30°C. They further investigated that the higher thermostability of L‐LDHs compared with that of D‐LDHs may be a major reason why the enantiopurity of d‐lactate is decreased at high fermentation temperatures (Gu et al., 2014).

Next, the kinetic parameters of the three D‐LDHs and two L‐LDHs of *S. inulinus* YBS1‐5 were determined at their respective optimal pH and temperature (Table 1). The apparent *K*_m_ of D‐LDH1 was lower than that of D‐LDH2 and D‐LDH3, indicating that D‐LDH1 had a higher affinity to pyruvate than D‐LDH2 and D‐LDH3. Furthermore, the *K*_m_ values of L‐LDH1 and L‐LDH2 were determined to be 4.1 and 6.8 mM, respectively, which was higher than those of three D‐LDHs. Therefore, D‐LDHs of *S. inulinus* YBS1‐5 had a higher affinity for pyruvate than L‐LDHs. Furthermore, D‐LDH1 exhibited approximately 5‐fold and 46‐fold higher catalytic efficiency (*k*_cat_/*K*_m_) than D‐LDH2 and D‐LDH3, suggesting that D‐LDH1 had a higher potential to efficiently produce d‐lactate from pyruvate than D‐LDH2 and D‐LDH3 in *S. inulinus* YBS1‐5. As expected, the two L‐LDHs showed low catalytic efficiency with pyruvate as the substrate.

**Table 1:** Specific activities and kinetic parameters of purified heterologously expressed *Sporolactobacillus inulinus* YBS1‐5 D‐LDHs and L‐LDHs for the conversion of pyruvate to lactate

Parameter	D‐LDH1	D‐LDH2	D‐LDH3	L‐LDH1	L‐LDH2
*K*_m_ (mM)	0.47 ± 0.04	1.4 ± 0.09	3.1 ± 0.08	4.1 ± 0.12	6.8 ± 0.19
*V*_max_ (U/mg)	62.7 ± 3.2	38.1 ± 2.2	9.2 ± 0.7	5.2 ± 0.5	1.9 ± 0.5
*k*_cat_ (s^−1^)	75.8 ± 0.3	44.7 ± 0.3	10.7 ± 0.2	5.9 ± 0.1	2.3 ± 0.1
*k*_cat_/*K*_m_ (mM^−1^ s^−1^)	161 ± 6	32 ± 2	3.5 ± 0.2	1.4 ± 0.1	0.3 ± 0.1

Notes

A kinetic study of D‐LDH1 was performed at pH 6.0 and 35°C. For D‐LDH2 and D‐LDH3, the assay pH was 7.5 and 5.5, respectively, whereas the assay temperature was 30°C. The kinetic parameters of L‐LDH1 and L‐LDH2 were determined at pH 7.0 at 45°C and 40°C, respectively.

**Figure 2:**
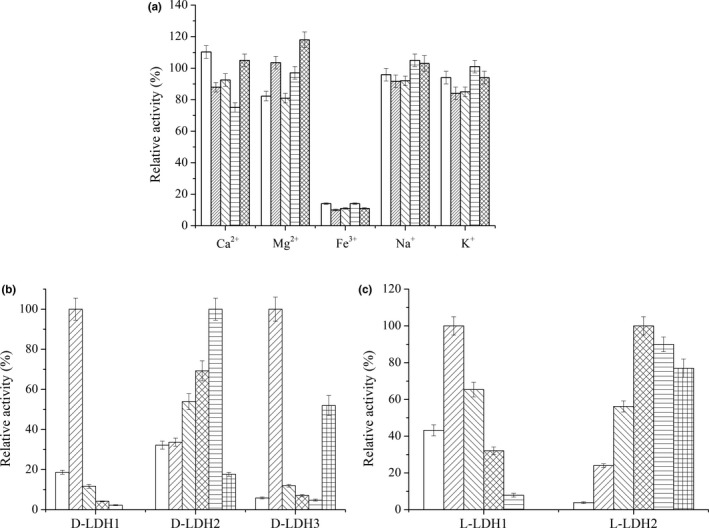
Effect of different metal ions on the activity of D‐LDHs and L‐LDHs (a) and substrate specificity of D‐LDHs (b) and L‐LDHs (c). Symbols represent: (a), D‐LDH1 (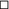), D‐LDH2 (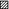), D‐LDH3 (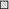), L‐LDH1 (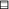), L‐LDH2 (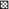). The concentration of metal ions was 5 mmol/L. The relative activity was calculated using the sample without metal ion as 100%. (b and c), glyoxylate (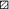), pyruvate (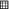), 2‐ketobutyrate (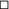), 2‐ketovalerate (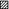), 2‐ketoisocaproate (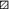) and phenylpyruvate (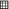).The relative activity of different LDHs toward tested substrate with the highest activity was defined as 100%, respectively. Errors bars represent the standard deviations of the means from three independent experiments

The effects of various metal ions on LDHs activity were tested (Figure 2a). Among the tested metal ions, Ca^2+^ displayed activation capability for D‐LDH1 activity, but inhibited L‐LDH1 activity. Mg^2+^ exhibited a slightly inhibitory effect on the activities of D‐LDH1 and D‐LDH3 while it activated L‐LDH2. However, no significant influence on LDH activity was observed with Na^+^ and K^+^. In general, divalent metal ions have diverse effects on the catalytic activity of LDHs from different bacterial sources. In a study by Furukawa, Miyanaga, Togawa, Nakajima, and Taguchi (2014), Ca^2+^ was shown to have a significant activation effect on *Pseudomonas aeruginosa* D‐LDH, whereas having no influence on *Fusobacterium nucleatum* D‐LDH and *E. coli* D‐LDH (). Furthermore, Mg^2+^ activated *F*. *nucleatum* D‐LDH and *P. aeruginosa* D‐LDH, but was unable to activate *E. coli* D‐LDH (Furukawa et al., 2014). Jiang et al., 2014 reported that Ca^2+^ activated *B*. *coagulans* L‐LDH at the highest degree. However, Gao et al., 2015 found that Mg^2+^ exhibited the highest activation capacity for *Pseudomonas stutzeri* L‐LDH activity.

The substrate specificity of D‐LDHs and L‐LDHs was analyzed with a variety of 2‐ketocarboxylic acids as presented in Figure 2b and c. D‐LDH1 exhibited narrow substrate specificity toward pyruvate among 2‐ketocarboxylic acid substrates, although it also showed a few catalytic activities toward glyoxylate and 2‐ketobutyrate. D‐LDH3 had substrate specificity similar to that of D‐LDH1, exhibiting the highest catalytic activity toward pyruvate. However, this enzyme also exhibited relatively high catalytic activity toward phenypyruvate. Zhu et al. (2015) also reported that d‐lactate dehydrogenase DLDH744 from *S. inulinus* had a high catalytic activity toward phenypyruvate. In their studies, the apparent *K*_m_ value for phenypyruvate was very close to that for pyruvate. In contrast to D‐LDH1 and D‐LDH3, D‐LDH2 displayed a broad substrate specificity toward relative bulky 2‐ketocarboxylic acids, and exhibited the highest catalytic activity toward 2‐ketoisocaproate. The d‐lactate dehydrogenases from *Leuconostoc mesenteroides* ATCC 8293, *P. aeruginosa* JCM8532 and *L. jensenii* SJ‐7A‐LIS were also reported to have narrow substrate specificities (Furukawa et al., 2014; Li et al., 2012), whereas d‐lactate dehydrogenases from *F. nucleatum* ATCC25586 and *L. jensenii* 269‐3 exhibited relative broad substrate specificities (Furukawa et al., 2014; Jun et al., 2013). Among L‐LDHs, L‐LDH1 showed the highest catalytic activity toward pyruvate among the tested 2‐ketocarboxylic acids, whereas L‐LDH2 exhibited high activity to pyruvate analogs with larger groups, such as 2‐ketovalerate and 2‐ketoisocaproate. *B. coagulan*s NL01 L‐LDH was also reported to have the highest activity toward pyruvate, but only a slight activity toward 2‐ketobutyrate and phenylpyruvate (Jiang et al., 2014).

### 3.3 Phylogenetic relationship of D‐LDHs and L‐LDHs from *S. inulinus* YBS1‐5

**Figure 3:**
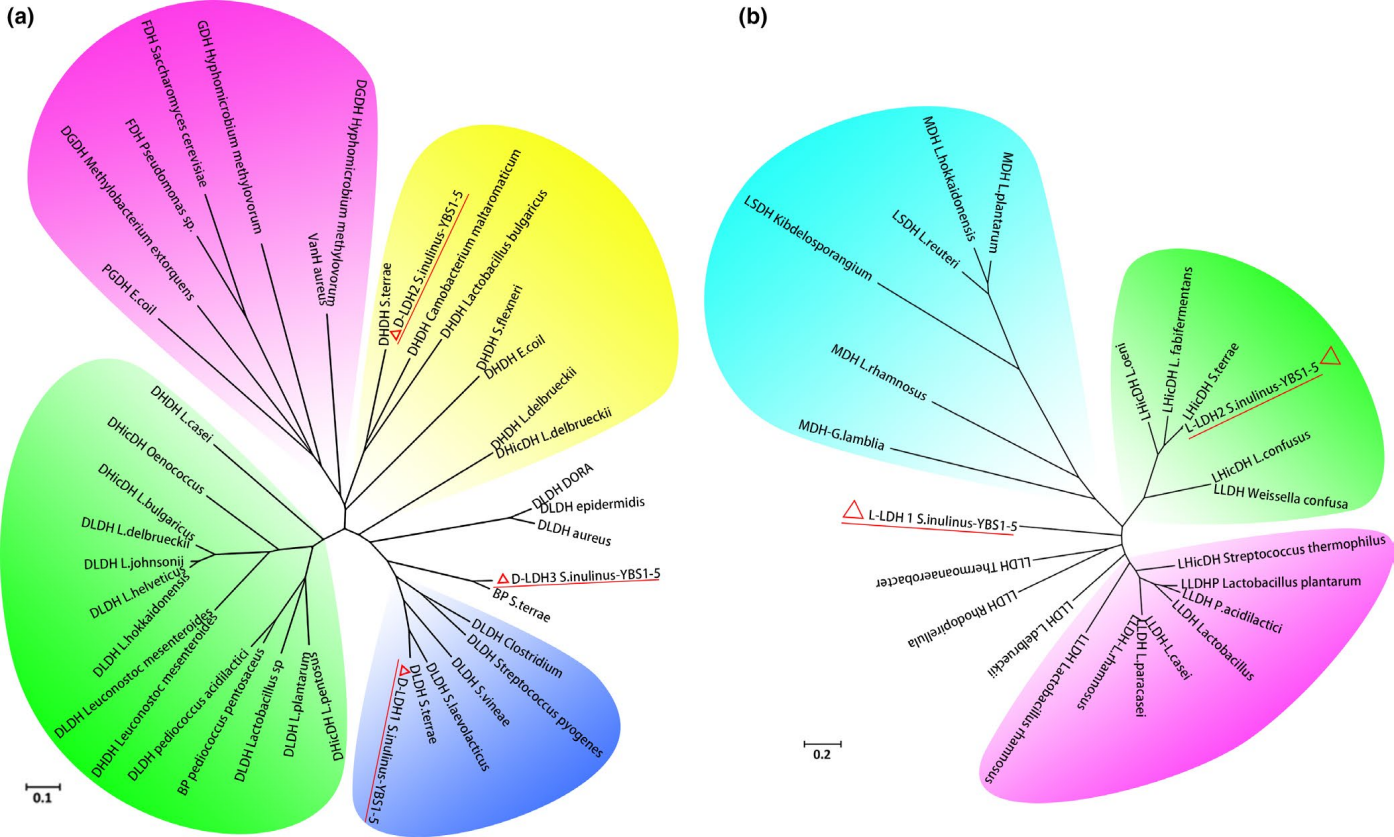
Phylogenetic tree of D‐LDHs (a) and L‐LDHs (b) with other enzymes in D‐2‐hydroxyacid dehydrogenase family or L‐2‐hydroxyacid dehydrogenase family from various microorganisms. The tree was constructed by NJ method

Previous studies demonstrated that D‐LDHs and L‐LDHs displayed significant differences in their amino acid sequences and were evolutionary unrelated (Cristescu & Egbosima, 2009; Jun et al., 2013; Wang et al., 2014; Zheng et al., 2012; Zhu et al., 2015). Therefore, we constructed two phylogenetic trees to analyze evolutionary relationships of D‐LDHs and L‐LDHs from *S. inulinus* YBS1‐5, respectively. Overall, D‐LDHs clustered with a large D‐2‐hydroxyacid dehydrogenase superfamily, comprised of various kinds of D‐isomer dehydrogenases such as d‐lactate dehydrogenase, D‐glycerate dehydrogenase, D‐phosphoglycerate dehydrogenase, D‐hydroxyglutarate dehydrogenase, D‐hydroxyisocaproate dehydrogenase, phosphite dehydrogenase, formate dehydrogenase, phenylpyruvate reductase, and 2‐ketopantoate reductase (Cristescu & Egbosima, 2009; Furukawa et al., 2014; Zhu et al., 2015). Although these enzymes possess highly divergent primary sequences, the core folding is structurally conserved, and the chiral product of the reaction is D‐isomer. In this study, a series of additional D‐2‐hydroxyacid dehydrogenases from different species were incorporated into phylogenetic analysis based on a keyword search and BLAST search (Table S2). The NJ tree showed that the *S. inulinus* YBS1‐5 D‐LDHs were clustered in three monophyletic groups (Figure 3a), consistent with the low sequence similarities of these D‐LDHs. The results suggested that three D‐LDHs were paralogs. D‐LDH1 was clustered into a clade with five other D‐LDHs mainly derived from *Sporolactobacillus* sp., and had the closest phylogenetic relationship with *S. terrae* D‐LDH. These D‐LDH‐producing strains were previously characterized as having a high d‐lactate production capacity (Li et al., 2013; Wang et al., 2011). It is worth noting that D‐LDH1 has low sequence similarity with other known D‐LDHs from *L. bulgaricus*,* L. delbrueckii,* and *L. casei,* and clusters into different groups. Cluster II that contained D‐LDH2 formed a monophyletic group with enzymes from *S*. *terrae*,* S. inulinus* YBS1‐5, *L. bulgaricus*, and *L. delbrueckii*. *L. delbrueckii* 2‐hydroxyacid dehydrogenase in this cluster was more phylogenetically close to D‐hydroxyisocaproate dehydrogenase and was active for a wide variety of 2‐oxoacid substrates (Bernard et al., 1994). In addition, *P. aeruginosa* PdxB in this cluster was a D‐erythronate‐4‐phosphate dehydrogenase with the biologically active form of vitamin B6 (Ha et al., 2007). The results indicated that D‐LDH2 might have substrate patterns different from those of typical D‐LDHs, with broader substrate spectrum compared with D‐LDH1. D‐LDH3 showed 100% sequence identity with DLDH744, which possessed both d‐lactate dehydrogenase and glutamate dehydrogenase activities, and was more phylogenetically close to D‐hydroxyisocaproate dehydrogenase (Zhu et al., 2015). The topology of the phylogenetic clustering of three D‐LDHs from *S. inulinus* YBS1‐5 was consistent with their variations in catalytic performance toward pyruvate (see above).

In the case of L‐LDHs, a BLAST search was also performed using L‐LDH1 and L‐LDH2 as templates. A series of other L‐LDHs, malate dehydrogenases, and L‐hydroxyisocaproate dehydrogenases from different species were selected to construct the phylogenetic tree of L‐LDHs (Figure 3b). A multiple sequence alignment illustrated that the similarity between L‐LDH1 and L‐LDH2 was only 31%. Consistently, these two L‐LDHs were clustered in different phylogenetic groups in the tree. In addition, both L‐LDH1 and L‐LDH2 were far from L‐LDHs of common l‐lactate‐producing strains such as *L. delbrueckii*,* L. lactis,* and *B. coagulans*. The L‐LDH phylogenetic tree indicated that L‐LDH1 was close to malate dehydrogenase from *G. lambila* and *L. rhamnosus*, whereas L‐LDH2 was closely related to *L. confuses* L‐hydroxyisocaproate dehydrogenase, which utilized a remarkably wide range of 2‐ketocarboxylic acids, with highest activities toward long chain C5‐ and C6‐ acids (Bao, Chatterjee, Lohmer, & Schomburg, 2007). This analysis was consistent with studies on the substrate specificity of L‐LDH2, which exhibited high activity toward 2‐ketocarboxylic acids with larger groups. This topology indicated that pyruvate was not the optimum substrate for L‐LDH1 and L‐LDH2, consistent with the low catalytic activities of two enzymes toward this substrate.

### 3.4 Changes in optical purity of d‐lactate under different neutralizing agents during fermentation

In d‐lactate production, the neutralizer plays a key role for cell growth, d‐lactate production, and purification (Nakano, Ugwu, & Tokiwa, 2012; Zheng et al., 2017). Indeed, multiple previous studies indicated that the final optical purity of d‐lactate was affected by the neutralizer employed in the reaction. In this context, the effects of neutralizing agents on the yield and optical purity of d‐lactate produced by *S. inulinus* YBS1‐5 were examined. CaCO_3_ was the most commonly used neutralizer for lactate production, as a slow and mild neutralizer only controlling the pH around 5.0. However, the optimal pH for D‐LDH1, D‐LDH2, and D‐LDH3 were 6.5, 7.5, and 5.5, respectively (Figure 1a). In addition, our previous studies indicated that the productivity, yield, and optical purity of d‐lactate all increased with increasing pH up to 6.5 when NaOH was used as the neutralizer for *S. inulinus* YBS1‐5 fermentation (Zheng et al., 2017). The above results suggested the acidic environment might thus be detrimental for several of the key enzymes, cell growth, and d‐lactate production. Therefore, the broth pH was maintained at 6.5 using NaOH, KOH, or NH_4_OH. When NaOH was used as a neutralizer, d‐lactate titer, and optical purity were 110.8 g/L and 98.1% after 68 hr of incubation. With KOH and NH_4_OH as neutralizers, optical purity of d‐lactate was only 93.7% and 91.3%, respectively. During fermentation with CaCO_3_ supplementation, the optical purity of product was 99.5% (Table 2). Notably, when NaOH was used as a neutralizer, the fermentation time was greatly shortened by about 28 hr compared with CaCO_3_ used. Meanwhile, with increased total broth volume by the addition of bulk neutralizer NaOH solution (about 0.5 L), the final d‐lactate yield was close to that obtained with CaCO_3_ as neutralizer. On the other hand, the use of CaCO_3_ led to the generations of by‐product CaSO_4_ during the subsequent separation of d‐lactate, approximately 1 ton of CaSO_4_ was produced for every ton of d‐lactate generated, and the disposal of CaSO_4_ posed considerable economic and ecological problems (Zheng et al., 2017). Unfortunately, in this case, the optical purity of d‐lactate was lower than that obtained from CaCO_3_. Thus, a study of the crucial factors affecting the optical purity of d‐lactate is critical.

**Table 2:** Effects of different neutralizers on fermentation of D‐lactate by *S. inulinus* YBS1‐5

Parameter	Alkaline neutralizer			
	CaCO_3_	NaOH	KOH	NH_4_OH
Fermentation Time (h)	96	68	68	68
d‐lactate titer (g/L)	125 ± 8	111 ± 6	95 ± 6	93 ± 6
l‐lactate titer (g/L)	0.4 ± 0.1	1.1 ± 0.1	3.1 ± 0.2	4.3 ± 0.2
Optical purity (%)	99.5	98.1	93.7	91.3

### 3.5 Changes in *ldh*Ds and *ldh*Ls transcription levels in the presence of different neutralizers

**Figure 4:**
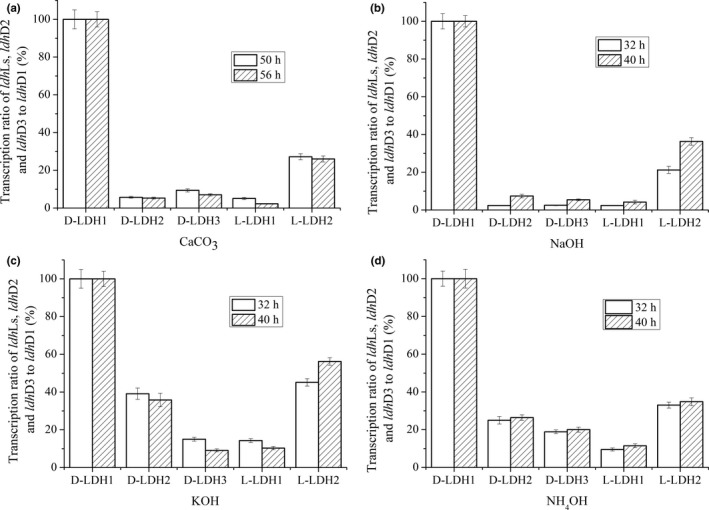
Determination of *ldh*Ds and *ldh*Ls transcription levels in *S. inulinus* YBS1‐5 under different neutralizers. a‐d Neutralized by CaCO3 (a), NaOH (b), KOH (c), NH4OH (d), respectively. Errors bars represent the standard deviations of the means from three independent experiments

To further explore the mechanisms of different neutralizers on optical purity of d‐lactate produced by *S. inulinus* YBS1‐5, quantitative real‐time (RT) PCR was employed to analyze associations of changes in optical purity with transcription of genes encoding key enzymes involved in d‐lactate formation. Although optically pure lactate was synthesized from pyruvate by catalysis of chirally specific D‐ or L‐LDHs, transcriptional analysis of *ldh*Ds and *ldh*Ls in *S. inulinus* has not been reported. Previous studies showed that *ldh*Ds transcription levels were higher than those of *ldh*Ls among various *Lactobacillus* strains, even for l‐lactate producers (Zheng et al., 2012). The distinct catalytic activities of L‐ and D‐LDHs contributed to differences in the ratio of the two isomers and different types of *Lactobacillus* strains. In contrast to the situation in *Lactobacillus* strains, the *ldh*L transcription level in *B. coagulans* 2‐6 was much higher than *that* of *ldh*D in all growth phases, which might explain high optical purity of the l‐lactate produced by this strain (Sun et al., 2016; Wang et al., 2014). Therefore, the crucial factors affecting the optical purity of lactate may differ among various LAB. According to previous reports on *B. coagulans* 2‐6, *L. delbrueckii* DSM 20081, and *L. plantarum* DSM 20714, the transcription levels of nLDH encoding genes were high in the exponential phase, which correlated with the marked change in the optical purity of lactate in the exponential phase cells (Wang et al., 2014). Therefore, in this study, *S. inulinus* YBS1‐5 cells at exponential phase were collected for transcription level measurements (Figure 4). With CaCO_3_ as a neutralizer, the *ldh*D1 transcription level was highest, and the transcription ratios of *ldh*L2 to *ldh*D1 were 27.2% and 26.0% at two different time points in the logarithmic phase (50 and 56 hr, respectively). By comparison, *ldh*D2, *ldh*D3m and *ldh*L1 transcription levels were rather low. When NaOH was used as the neutralizer, the transcription ratio of *ldh*L1 to *ldh*D1 slightly increased from 2.3% (CaCO_3_ as the neutralizer) to 4.2% after 40 hr of incubation, whereas the transcription ratio of *ldh*L2 to *ldh*D1 increased from 26.0% to 36.2%. Correspondingly, the optical purity of d‐lactate slightly decreased from 99.5% to 98.1%. In the presence of KOH, the relative transcription levels of *ldh*D2, *ldh*L1, and *ldh*L2 were increased to 35.8%, 10.3%, and 56.2%, with no obvious change in *ldh*D3 transcription. For NH_4_OH, *ldh*D2, *ldh*D3, *ldh*L1, and *ldh*L2 transcription levels were all elevated in comparison to *ldh*D1. Thus, for each of four neutralizers used, *ldh*D1 transcription levels were the highest, consistent with highest catalytic activity of D‐LDH1 toward pyruvate. These findings indicated that D‐LDH1 played a central role in production of optically pure d‐lactate in *S. inulinus* YBS1‐5. In addition, when KOH and NH_4_OH were used as neutralizers, the significant increase in *ldh*L1 transcription was correlated with a marked reduction in optical purity of d‐lactate to 93.7% and 91.3%, which suggested that only L‐LDH1 was the key factor affecting optical purity of d‐lactate produced by *S. inulinus* YBS1‐5. In addition, many previous reports have revealed that l‐lactate permeases are involved in l‐lactate utilization in different strains (Gao et al., 2015; Jiang, Gao, Ma, & Xu, 2014). Guided by the whole‐genome sequence of *S. inulinus* CASD, we measured the transcription levels of l‐lactate permease‐encoding gene (*lld*P) under different neutralizers. The results indicated that the *lld*P transcription levels were rather low. With CaCO_3_ as the neutralizer, the transcription ratio of *ldh*D1 to *lld*P was approximate 800‐fold. Furthermore, no transcription was detected in *lld*P when NaOH, KOH or NH_4_OH was used as the neutralizer. It seemed that the contribution of l‐lactate permease to the optical purity of d‐lactate in *S. inulinus* YBS1‐5 appeared to be minimal. Ongoing studies in our laboratory assess the roles of the individual D‐ and L‐LDHs in growth and metabolism of strain YBS1‐5, as well as their transcriptional regulation.

## 4 CONCLUSION

In conclusion, three D‐LDH‐ and two L‐LDH‐encoding genes in *S. inulinus* YBS1‐5 were cloned, expressed, and characterized. The effects of different neutralizers on transcription levels of five genes and optical purity of d‐lactate were assessed. The high catalytic efficiency of D‐LDH1 toward pyruvate and elevated *ldh*D1 transcription level suggest that D‐LDH1 plays a central role in d‐lactate production by *S. inulinus* YBS1‐5. In addition, it has become apparent that L‐LDH1 mostly contributes to optical purity of d‐lactate. These findings may provide useful guidance for further strain improvements and polymer‐grade d‐lactate production.

## CONFLICT OF INTEREST

The authors declare no financial or commercial conflict of interest.

## Data Availability

All data generated or analyzed during this study are included in this published article.
