# Supplementary material for: Relative catalytic efficiencies and transcript levels of three d‐ and two l‐lactate dehydrogenases for optically pure d‐lactate production in Sporolactobacillus inulinus
Source: Microbiologyopen. 2018 Aug 1;8(5):e00704. doi: 10.1002/mbo3.704 (PMC6528580; doi:10.1002/mbo3.704)
Supplement: Supplementary file 1 [file MBO3-8-e00704-s001.doc]

**Table S1.** Primers for amplification and expression of *ldh*Ds and *ldh*Ls in *S. inulinus* YBS1-5

| Primers | Sequences (5’ to 3’) | Description |
| --- | --- | --- |
| D-LDH1-F1 | ATGAAGCTATTCATGTATGGTGTCC | Primers for amplification of *ldh*D1 |
| D-LDH1-R1 | TTATTGAGTGACAGCCGGCTTC |
| D-LDH2-F1 | ATGGCTTTTAAAATTATTGCG | Primers for amplification of *ldh*D2 |
| D-LDH2-R1 | TCATTTTTTTGCTGGTTC |
| D-LDH3-F1 | ATGAAAATCATTATGTTCAGCGT | Primers for amplification of *ldh*D3 |
| D-LDH3-R1 | TTAGTTTTCTACAGCTACTTTGTTC |
| L-LDH1-F1 | GTGAGCGAAACAGTCAGACGAG | Primers for amplification of *ldh*L1 |
| L-LDH1-R1 | TCAGTTTGCGGTAGTTTCTTCG |
| L-LDH2-F1 | ATGAGAAAGTATGCGATTATTGGTC | Primers for amplification of *ldh*L2 |
| L-LDH2-R1 | TCAGTTGGTTTGTTTTTCAAACGTT |
| D-LDH1-F | GCGGGCTAGCGACGACGACGACAAGATGAAGCTATTCATGTATGGTGTCC (*Nhe* I) | Primers for expression of *ldh*D1 |
| D-LDH1-R | GCGGAAGCTTTTATTGAGTGACAGCCGGCTTC (*Hind* III) |
| D-LDH2-F | GCGGCATATGGACGACGACGACAAGATGGCTTTTAAAATTATTGCG (*Nde* I) | Primers for expression of *ldh*D2 |
| D-LDH2-R | GCGCAAGCTTTCATTTTTTTGCTGGTTC (*Hind* III) |
| D-LDH3-F | GCGGCATATGGACGACGACGACAAGATGAAAATCATTATGTTCAGCGT (*Nde* I) | Primers for expression of *ldh*D3 |
| D-LDH3-R | GCGGAAGCTTTTAGTTTTCTACAGCTACTTTGTTC (*Hind* III) |
| L-LDH1-F | GCGGCATATGGACGACGACGACAAGGTGAGCGAAACAGTCAGACGAG (*Nde* I) | Primers for expression of *ldh*L1 |
| L-LDH1-R | GCGGAAGCTTTCAGTTTGCGGTAGTTTCTTCG (*Hind* III) |
| L-LDH2-F | GCGGCATATGGACGACGACGACAAGATGAGAAAGTATGCGATTATTGGTC (*Nde* I) | Primers for expression of *ldh*L2 |
| L-LDH2-R | GCGGAAGCTTTCAGTTGGTTTGTTTTTCAAACGTT (*Hind* III) |

**Table S2** Sequences included in phylogenetic analyses of D-lactate dehydrogenase

| Assignment | Organisms | Accession number |
| --- | --- | --- |
| DLDH1 *S. inulinus* YBS-15 | *Sporolactobacillus inulinus* YBS1-5 | This study |
| DLDH *S. terrae* | *Sporolactobacillus* *terrae* | WP_028976598.1 |
| DLDH *S. epidermidis* | *Staphylococcus epidermidis* | WP_049371672.1 |
| DLDH *S.pyogenes* | *Streptococcus pyogenes* | Q99ZM2 |
| DLDH *S. laevolacticus* | *Sporolactobacillus laevolacticus* | WP_023511201.1 |
| DLDH *Clostridium* | *Clostridium mangenotii* | WP_027701643.1 |
| DLDH *L.bulgaricus* | *Lactobacillus delbrueckii* 2038 | ADY84251.1 |
| DLDH *S.* DORA | *Staphylococcus sp.* DORA_6_22 | ETJ13655.1 |
| DLDH *L.mesenteroides* | *Leuconostoc mesenteroides* subsp. | P51011.1 |
| DLDH *Lactobacillus* | *Lactobacillus sp.* MONT4 | AAR19203.1 |
| DLDH *L. helveticus* | *Lactobacillus helveticus* | 2109198A |
| DLDH *L. johnsonii* | *Lactobacillus johnsonii* | AAC99363 |
| DLDH *L.delbrueckii* | *Lactobacillus delbrueckii* | CAA42781 |
| DLDH *L.helveticus* | *Lactobacillus helveticus* DSM 20075 | EEW68509.1 |
| DLDH *P. acidilactici* | *Pediococcus acidilactici* | CAA50275 |
| DLDH *L. plantarum* | *Lactobacillus plantarum* | BAA14352 |
| DLDH *S. aureus* | *Staphylococcus aureus* | AAB17663 |
| DLDH *S. vineae* | *Sporolactobacillus vineae* | WP_010632485.1 |
| DHDH2 *S. inulinus* YBS1-5 | *Sporolactobacillus inulinus* YBS1-5 | This study |
| DHDH *L.bulgaricus* | *Lactobacillus delbrueckii* 2038 | ADY85822.1 |
| DHDH *E. coli* | *Escherichia Coli* BL21(DE3) | 3WX0_A |
| DHDH *S. flexneri* | *Shigella flexneri* 1485-80 | EJZ67254.1 |
| DHDH *L. casei* | *Lactobacillus casei* | AAA25236.1 |
| DHDH *L. mesenteroides* | *Leuconostoc mesenteroides* | P51011.1 |
| DHDH *L. delbrueckii* | *Lactobacillus delbrueckii* | CAA46324.1 |
| DHDH *C. maltaromaticum* | *Carnobacterium maltaromaticum* | WP_015075117.1 |
| DHDH *S. terrae* | *Sporolactobacillus terrae* | WP_028977125.1 |
| DLDH3 *S. inulinus* YBS1-5 | *Sporolactobacillus inulinus* YBS1-5 | This study |
| DLDH *S. terrae* | *Sporolactobacillus* *terrae* | WP_028983792.1 |
| DLDH *P. pentosaceus* | *Pediococcus pentosaceus* | WP_011673315.1 |
| DHicDH *Oenococcus* | Oenococcus oeni DSM 20252 | EKP90311.1 |
| DHicDH *L. pentosus* | *Lactobacillus pentosus* | P26298.1 |
| DHicDH *L. delbrueckii* | *Lactobacillus delbrueckii* | Q48534 |
| DHicDH *L. delbrueckii* | *Lactobacillus delbrueckii* ATCC 11842 | P26297.3 |
| FDH *Pseudomonas* | *Pseudomonas* sp. 101 | P33160 |
| FDH *S. cerevisiae* | *Saccharomyces cerevisiae* | Q08911 |
| VanH *E. faecium* | *Enterococcus faecium* | Q05709 |
| VanH *S. aureus* | *Staphylococcus aureus* | Q7BWD8 |
| DGDH *H. methylovorum* | *Hyphomicrobium methylovorum* | P36234 |
| DGDH *M. extorquens* | *Methylobacterium extorquens* | WP_003597638.1 |
| PGDH *Escherichia. coli* | *Escherichia. Coli* | P0A9T0 |

**Table S3** Sequences included in phylogenetic analyses of L-lactate dehydrogenase

| Assignment | Organisms | Accession number |
| --- | --- | --- |
| LLDHP *L. plantarum* | *Lactobacillus plantarum* | AIC33511.1 |
| LLDH1 *S. inulinus YBS1-5* | *Sporolactobacillus inulinus* YBS1-5 | This study |
| LLDH *L. rhamnosus* | *Lactobacillus rhamnosus Lc 705* | CAR89424.1 |
| LLDH *Lactobacillus sp.* | *Lactobacillus vini* | WP_010580386.1 |
| LLDH *T. ethanolicus* | *Thermoanaerobacter ethanolicus* | WP_003870794.1 |
| LLDH *Rhodopirellula* | *Rhodopirellula baltica* | WP_007324694.1 |
| LLDH *L. casei* | *Lactobacillus paracasei* | WP_003567646.1 |
| LLDH *P. acidilactici* | *Pediococcus acidilactici* | Q59645.1 |
| LLDH *L. delbrueckii* | *Lactobacillus delbrueckii* | WP_013438917.1 |
| LLDH *W. confuse* | *Weissella confuse* | WP_003607654.1 |
| LLDH2 *S. inulinus* YBS1-5 | *Sporolactobacillus inulinus* YBS1-5 | This study |
| LHicDH *S. terrae* | *Sporolactobacillus terrae* | WP_028975883.1 |
| LHicDH *L. fabifermentans* | *Lactobacillus fabifermentans* | WP_024625637.1 |
| LHicDH *L. oeni* | *Lactobacillus oeni* DSM 19972 | KRL05773.1 |
| LHicDH *S. thermophilus* | *Streptococcus thermophilus* | WP_011227325.1 |
| LHicDH *L. confuses* | *Weissella confusa* | WP_003607654.1 |
| MDH *L. rhamnosus* | *Lactobacillus rhamnosus* | WP_005689193.1 |
| MDH *L. hokkaidonensis* | *Lactobacillus hokkaidonensis* | KRO09472.1 |
| MDH *L. plantarum* | *Lactobacillus plantarum ATCC 14917* | EFK30005.1 |
| LSDH *Kibdelosporangium* | *Kibdelosporangium sp.* MJ126-NF4 | CTQ97072.1 |
| LLDH *L. casei* | *lactobacillus casei* | WP_003662706.1 |
| LLDH *L. rhamnosus* | *Lactobacillus rhamnosus* | WP_029943808.1 |
| MDH *G. lamblia* | *Giardia lamblia ATCC 50803* | XP_001707118.1 |
| LSDH *L. reuteri* | *Lactobacillus reuteri* | CUR38917.1 |

**Table S4.** Specific primers for quantitative real-time PCR.

| Target gene | Sequence (5’ to 3’) |
| --- | --- |
| *ldh*D1 | CGCACTCATTGATACCGAAGCCTT |
|  | AGGAGCACCTGATCCATAGCGATTA |
| *ldh*D2 | ACCACATTGATCTTCAGGCTGCT |
|  | TCGTGGCTGAAGTTTGTTTCTGTTA |
| *ldh*D3 | CCGCTTATGGATTCAACGACTCA |
|  | TTGAAGAAGTGCTCCTCACCGTT |
| *ldh*L1 | ATCCATACACGCAAAGGCAATACC |
|  | TCGCTGAGGCGGAACTTGATGA |
| *ldh*L2 | TGCGAAAGAACGGAACCTAAACC |
|  | GGCTGACCAACATAAGTGCCGTA |
| *lld*P | CGGCATTCCCATTCTCAC |
|  | TCGTTTCCATCCCGACATA |

**
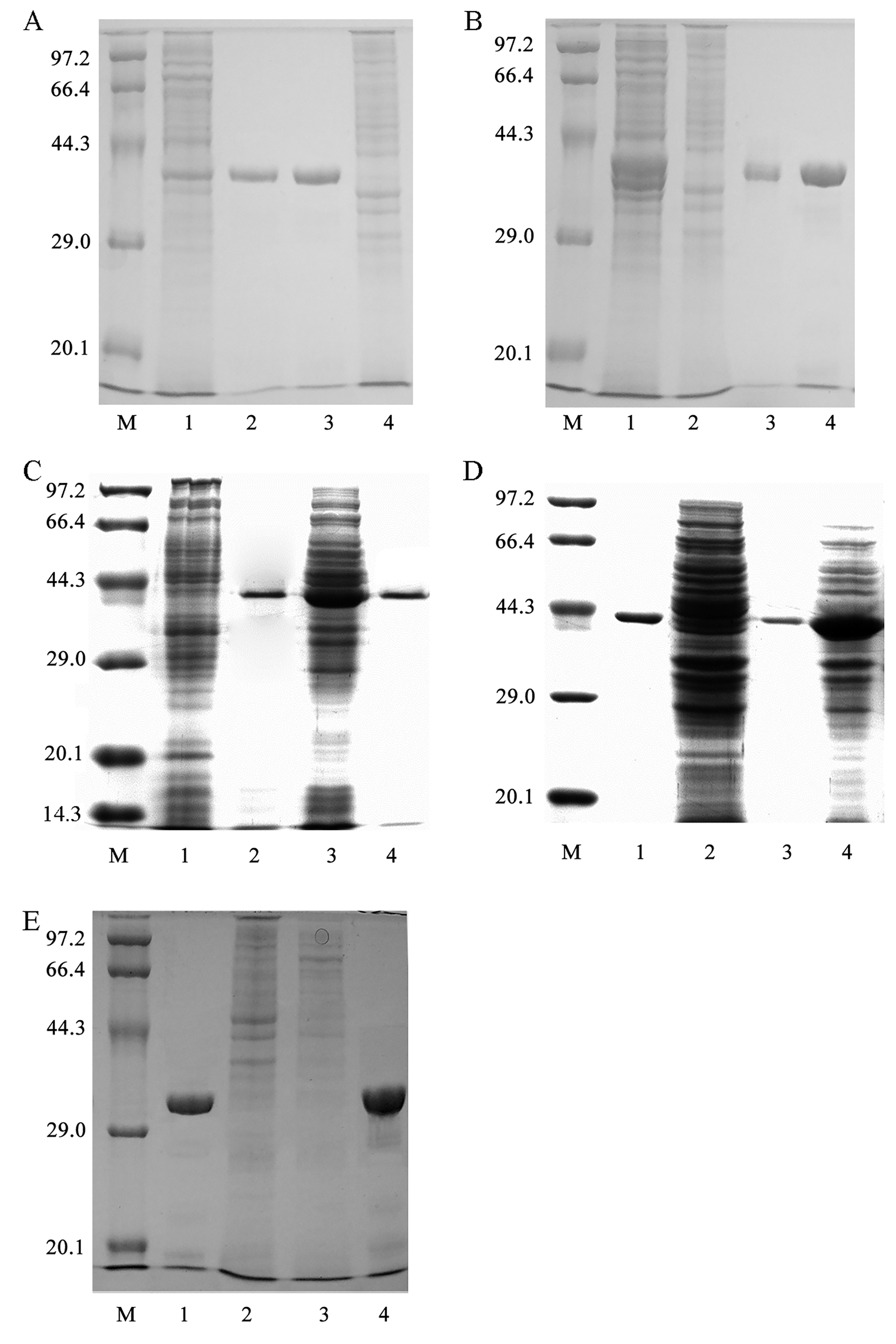
**

**Figure S1 SDS-PAGE of purified D-LDHs and L-LDHs.** (A) lane 1, crude extracts of BL21-pET-*ldh*D1; lane 2, purified D-LDH1 without His tag; lane 3, purified D-LDH1; lane 4, crude extracts of BL21-pET28a. (B) lane 1, crude extracts of BL21-pET-*ldh*D2; lane 2, crude extracts of BL21-pET28a; lane 3, purified D-LDH2 without His tag; lane 4, purified D-LDH2. (C) lane 1,crude extracts of BL21-pET28a; lane 2, purified D-LDH3; lane 3, crude extracts of BL21-pET-*ldh*D3; lane 4, purified D-LDH3 without His tag. (D) lane 1, purified L-LDH1; lane 2, crude extracts of BL21-pET28a; lane 3, purified L-LDH1 without His tag; lane 4, crude extracts of BL21-pET-*ldh*L1. (E) line 1, purified L-LDH2 without His tag; lane 2, crude extracts of BL21-pET-*ldh*L2; lane 3, crude extracts of BL21-pET28a; lane 4, purified L-LDH2. Lane M, protein marker.
